# Elbow arthroscopy in children and adolescents: analysis of outcome and complications

**DOI:** 10.1186/s40001-018-0338-5

**Published:** 2018-09-15

**Authors:** Jörg Nowotny, Sebastian Löbstein, Achim Biewener, Guido Fitze, Philip Kasten

**Affiliations:** 1 Orthopaedic–Traumatology Centre (OUC), Carl-Gustav Carus University, Dresden, Germany; 2 Paediatric Surgery, Carl-Gustav Carus University, Dresden, Germany; 3 Orthopaedic–Surgery Center (OCC), Tübingen, Germany

## Abstract

**Background:**

Chondral or osteochondral lesions, post-traumatic contracture and loose bodies of the elbow are often associated with chronic pain, stiffness, repetitive swelling and joint blockages. Therefore, arthroscopy of the elbow is often used in the elderly for the treatment of osteochondral defects or arthrolysis. There are only a few reports and studies about arthroscopic therapy of the elbow in children and adolescents. This study assesses the clinical outcome of arthroscopic therapy in this age group.

**Methods:**

In a retrospective study, children and adolescents who underwent an elbow arthroscopy in the period from 2010 to 2014 were included. The children were evaluated using the validated outcome measures Mayo Elbow Performance Score (MEPS), range of motion, pain on visual analog scale (VAS), Oxford Elbow Score (OES), quick dash and postoperative satisfaction. Furthermore, all complications were analyzed.

**Results:**

In total, 27 patients were included. The mean (range) age was 14 (11–17) years, with a follow-up of 45 months. Fourteen (52%) were female and thirteen children (48%) were male. Twenty children had an arthroscopy due to osteochondritis dissecans and seven children for post-traumatic pain and stiffness. The mean (standard deviation) MEPS improved from 65 (15) to 96 (8; *p* = .005). The OES and quick dash were 93 and 5.4. The mean extension improved from − 15° (± 13.8) to 3° (± 10.2; *p* < .001). The mean flexion improved from 131° (± 13.4) to 137° (± 9.5; *p* = .003). Average pain on VAS was postoperative .2 (± .5), and 81.5% of all children had excellent or good results. There were no complications such as damage of nerves or blood vessels observed.

**Conclusion:**

Elbow arthroscopy is an appropriate and safe treatment option in children and adolescents with good and excellent postoperative results.

## Background

Chondral or osteochondral lesions, post-traumatic scarring and loose bodies of the elbow are often associated with chronic pain, repetitive swelling and joint blockages. Besides the classic osteoarthritis of the elderly, often children and adolescents are affected. In particular, repetitive stress on the elbow, e.g., as part of professional sports, seems to play an essential role [11, 12], in which most common the capitellum and more rarely the trochlea are concerned [19].

In children, a trauma of the elbow joint, for example after diacondylar or radial head fracture with involvement of the joint, frequently causes osteochondral defects. Also dislocation or subluxation of the elbow can cause avulsions of the cartilage and subsequently post-traumatic osteoarthritis.

Besides, non-traumatic injuries play an important role. It is important to distinguish between Panner’s disease and osteochondritis dissecans (OCD). Panner’s disease was first described in 1927 by Hans Jessen Panner and often affects young males between the 7th and the 12th year of life. It is characterized by ischemia and necrosis of the epiphysis, mostly of the capitellum, without damaging the cartilage. In contrast, the OCD is an infraction of the subchondral bone and the overlying layer of cartilage, and can ultimately lead to the separation of an osteochondral fragment [21]. The reason appears to be repetitive microtrauma, overuse and ischemic processes (for example in young gymnasts or throwing athletes) and has potentially a genetic component [5, 18].

However, elbow cartilage damage can be asymptomatic due to the lack of load-bearing function at the initial stage. Further, in an advanced stage a lateral pain, limitation of range of motion (particularly extension deficit), swelling, blockages, cracking noises and secondary instabilities can be observed.

Primary imaging includes the radiograph of the elbow joint in the anterior–posterior (AP) and lateral path to detect OCD, Panner’s and Hegemann disease (aseptic necrosis of the trochlea) and potential loose bodies. For early assessment of the cartilage, magnetic resonance imaging (MRI) is the gold standard [8].

**Fig. 1:**
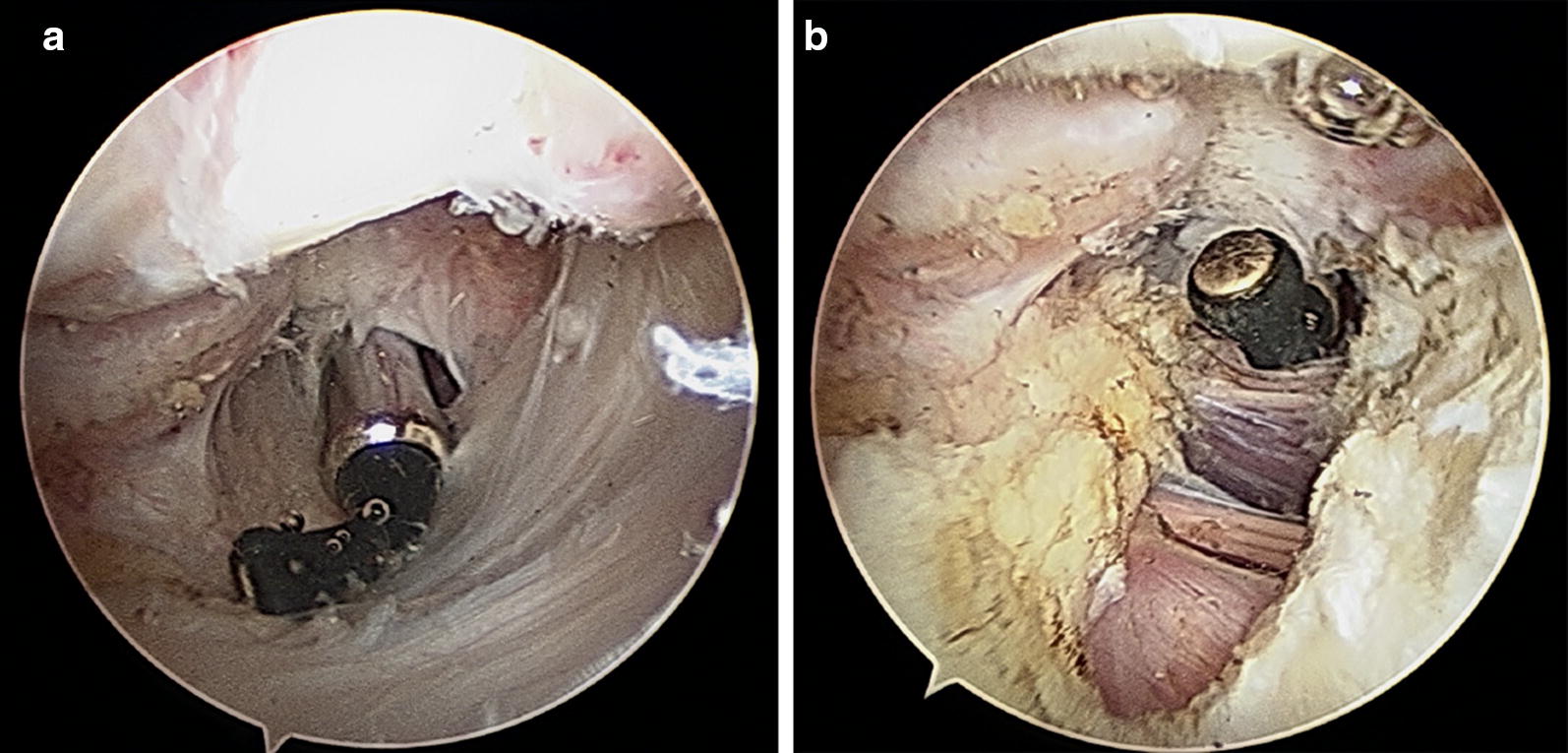
Treatment of extension deficit with anterior capsulotomy: **a** arthroscopic view of the ventral elbow compartment with hypertrophic ventral capsule, **b** after ventral capsulotomy by an ulnar approach

**Fig. 2:**
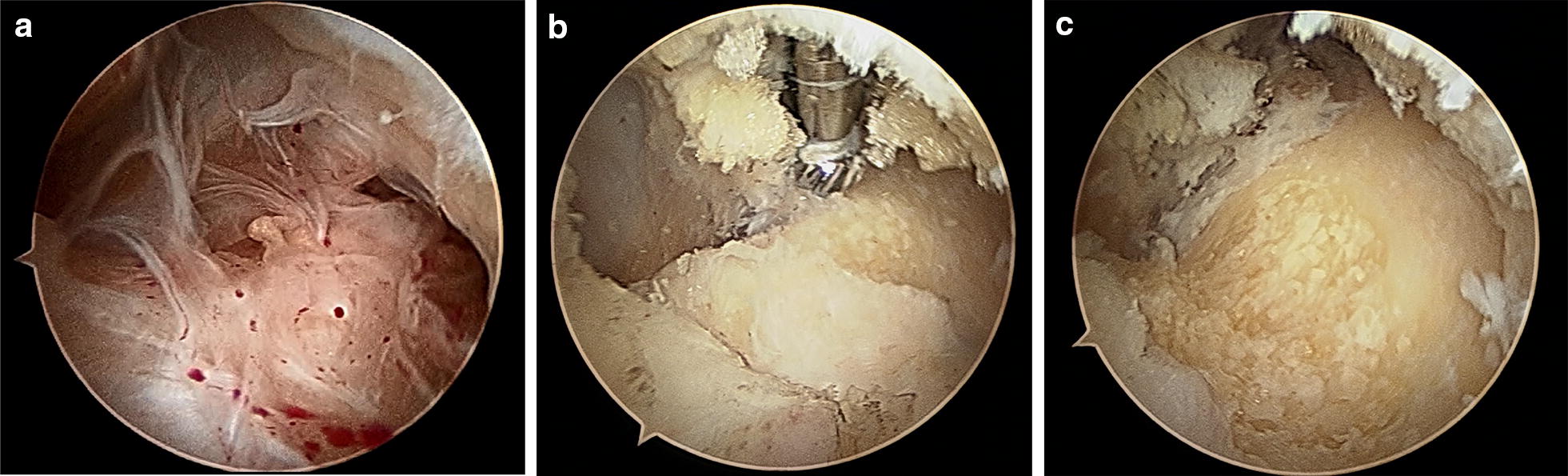
Treatment of extension deficit with fossa olecrani plastic/modified Outerbridge–Kashiwagi procedure: **a** arthroscopic view of a hypertrophic fossa olecani via a dorsal portal, **b** arthroscopic preparation with ball cutter, **c** restored fossa olecrani

While conservative treatment is indicated when the cartilage is intact (stable) and no loose bodies are visualized, a surgical treatment is feasible for persistent pain, unsuccessful conservative treatment in OCD lesion grades III and IV (Table 1), loose bodies and avulsion lesions. The choice of treatment depends on the genesis (degenerative or traumatic), the size of defect and thickness of the cartilage, localization and classification. In the case of reduced range of motion or existing stiffness of the elbow, an arthroscopic arthrolysis can be performed. There are various methods depending on the localization of the pathology and extent of the restriction such as anterior capsulotomy (Fig. 1) and/or modified Outerbridge–Kashiwagi procedure (Fig. 2).

**Table 1:** MRI classification of osteochondral lesions according to DiPaola et al. [6]

Grade	Findings in the MRI
I	No break in articular cartilage, thickening of articular cartilage
II	Articular cartilage breached, low signal rim behind fragment, indicating fibrous attachment
III	Articular cartilage breached with high signal T2 changes behind fragment suggesting fluid behind the lesion
IV	Loose bodies in the joint with lesion of the articular surface

Due to the development of operative procedures and technologies, as well as increasing clinical experience, elbow arthroscopy has become an established method in the diagnosis and treatment of elbow pathologies [4]. However, arthroscopic skills and good anatomical knowledge are necessary to avoid complications.

The aim of the present study is to evaluate the surgical procedure with regard to outcome and the safety of elbow arthroscopy in paediatric and adolescent patients with significant traumatic and non-traumatic joint pathology.

## Methods

### Study design

In a retrospective study, children were consecutive involved who underwent elbow arthroscopy between 2010 and 2014 at a University Center for Orthopedics and Traumatology. Approval for the study was granted by the local ethics committee (EK 433102016).

### Patients

In the observation period, 35 elbow arthroscopies in 32 children and adolescents were performed. From these 35 arthroscopies, 27 could be recruited and examined (dropout rate: 23%). The mean (range) age was 14 (11–17) years. The mean follow-up period was 45.6 months. Fourteen (52%) were female and thirteen children (48%) were male. In thirteen cases (48%), the right elbow was operated.

### Surgical technique

**Fig. 3:**
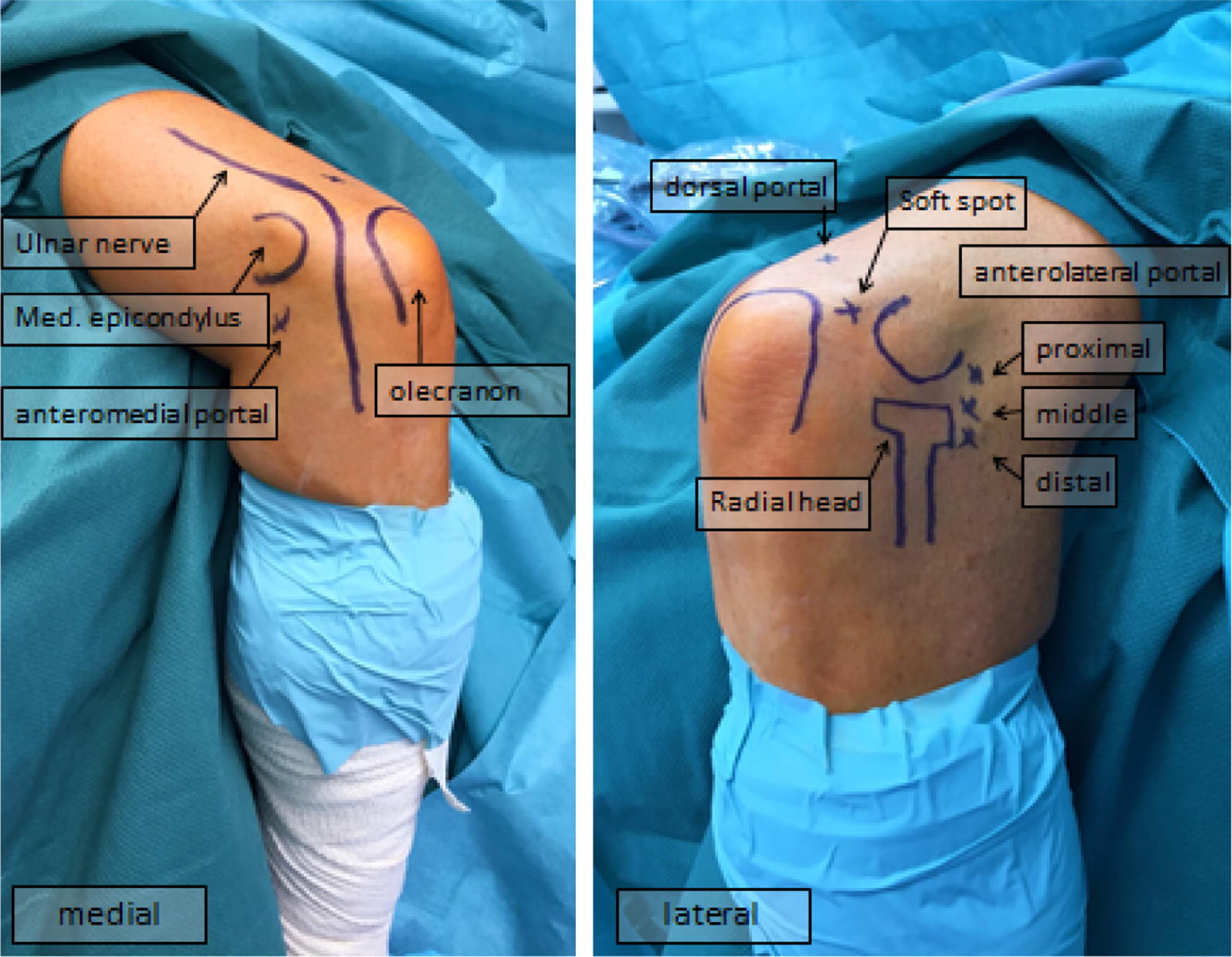
Standard portals for elbow arthroscopy

All procedures were performed under regional anesthesia (interscalene brachial plexus blockade) and/or in general total intravenous anesthesia. Three different surgeons carried out the operations, where as a surgeon did almost three quarters of all procedures. The positioning of the patient is important and can be done in back, lateral or ventral position [1, 17], whereas in the present study patients were treated in a lateral decubitus position using a tourniquet placed at the upper arm. Bony landmarks (radial head, epicondyles, olecranon process) were marked and the joint was distended with an intra-articular injection of 20–30 mL of saline solution through the soft spot. We used a 4 mm/30° arthroscope and a two roller-pump with low pressure of 30–40 mmHg. The standard portals such as the medial anterolateral, anteromedial, posterolateral, posterocentral and lower dorsolaterales portal were preferred (Fig. 3).

### Outcomes

The children were examined by one independent person who was not involved in surgery care using validated outcome measures such as range of motion (ROM) measured exact with a goniometer, Mayo Elbow Performance Score (MEPS), pain on visual analog scale (VAS), Oxford Elbow Score (OES), quick dash [10], postoperative satisfaction and reports on peri-/postoperative complications. The MEPS is defined in case of over 90 points as an excellent, between 75 and 89 as good, between 60 and 74 as fair and fewer than 60 as a poor result.

### Statistical analysis

For statistical analysis, SPSS Statistics software (version 20; IBM, Armonk, NY, USA) was used. In addition to the descriptive statistics, the differences between the preoperative and postoperative mean values were evaluated using Wilcoxon test for unpaired samples (significance level *p* < .05). All data are presented as mean with standard deviation.

## Results

### Patients and indication

20 children (74%) had arthroscopy due to an osteochondritis dissecans (OCD) with a grade III or IV according to DiPaola [6] and 7 children (26%) due to a past fracture of the elbow (PFE) and persistent complaints. The period from accident to surgery was in average 12 months in the PFE group and 6 months from beginning of symptoms to surgery in the OCD group. The operation was indicated due to isolate contracture in 3 (11%), contracture and pain in 7 (26%), loose bodies in 1 (4%), loose bodies and pain in 1 (4%), loose bodies, contracture and pain in 14 (51%), instability, contracture and pain in 1 (4%) of the cases.

### Analysis of intervention

In the group of OCD in 20% (*n* = 4) an arthrolysis, in 60% (*n* = 12) a synovectomy, in 75% (*n* = 15) a removal of loose bodies, in 25% (*n *= 5) a removal of an instable osteochondritis dissecans and in 40% (*n* = 8) a microfracture were performed. In the group of the PFE in 100 (*n* = 7) an arthrolysis (mostly with intra-articular debridement, anterior capsulotomy and/or modified Outerbridge–Kashiwagi procedure), in 86% (*n* = 6) a synovectomy and in 14% (*n* = 1) a removal of loose bodies were done.

### Analysis of outcome parameters

**Fig. 4:**
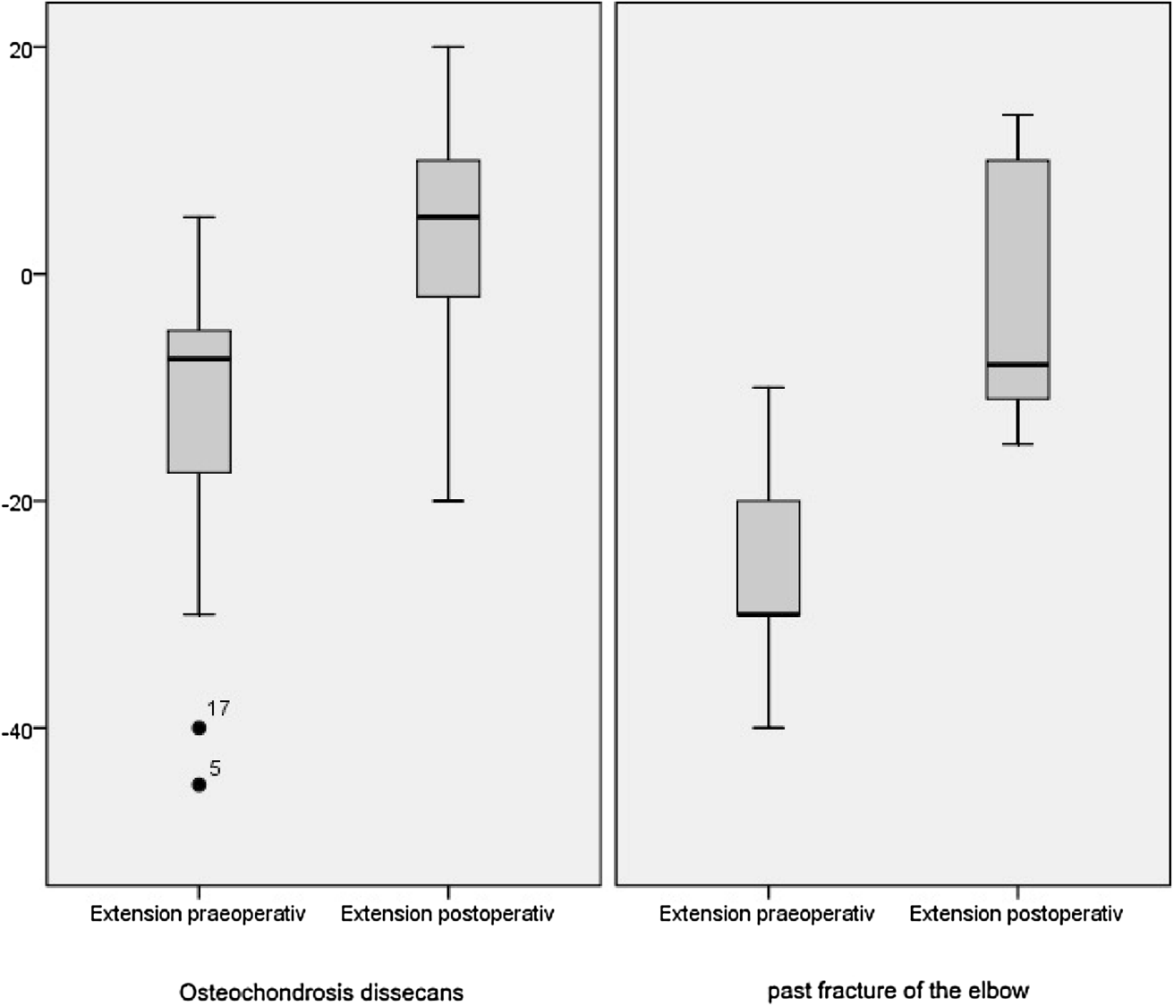
Boxplot of extension before and after intervention left: OCD group − 12° (SD: 13) to 4° (SD: 9), [*p* < .001]; right: arthrolysis group − 26° (SD: 10) to − 2° (SD: 12), [*p* = .018]

The overall range of motion (ROM) could be improved by the operative intervention in both groups. An overview is given in Table 2. The mean (standard deviation) extension increased significantly from − 15° (13.8) to 3° (10.2; *p* < .001) at the final follow-up. In the subgroups, the extension after the operation was in the group PFE less (− 2° ± 12) than in the OCD group (4° ± 9). However, in both groups a significant improvement in the extension could be achieved postoperatively (OCD: from − 12° [SD: 13] to 4° [SD: 9], *p* < .001; PFE: from − 26° [SD: 10] to − 2° [SD: 12], *p* = .018) (Fig. 4).

**Table 2:** Data baseline and outcome parameter

Score	osteochondritis dissecans group (*n* = 20)	SD	*p* value	Arthrolysis group (*n* = 7)	SD	*p* value
Age	14.3	2.3	–	13.4	1.9	–
Male	9	–	–	4	–	–
Improvement of extension in degree	16	12	< .001	24	18	.018
Improvement of flexion in degree	6	9	.018	9	8	.068
Improvement of MEPS	27	14	.027	38	17	.068
Postoperative pain on VAS	.25	.64	–	0	0	–

**Fig. 5:**
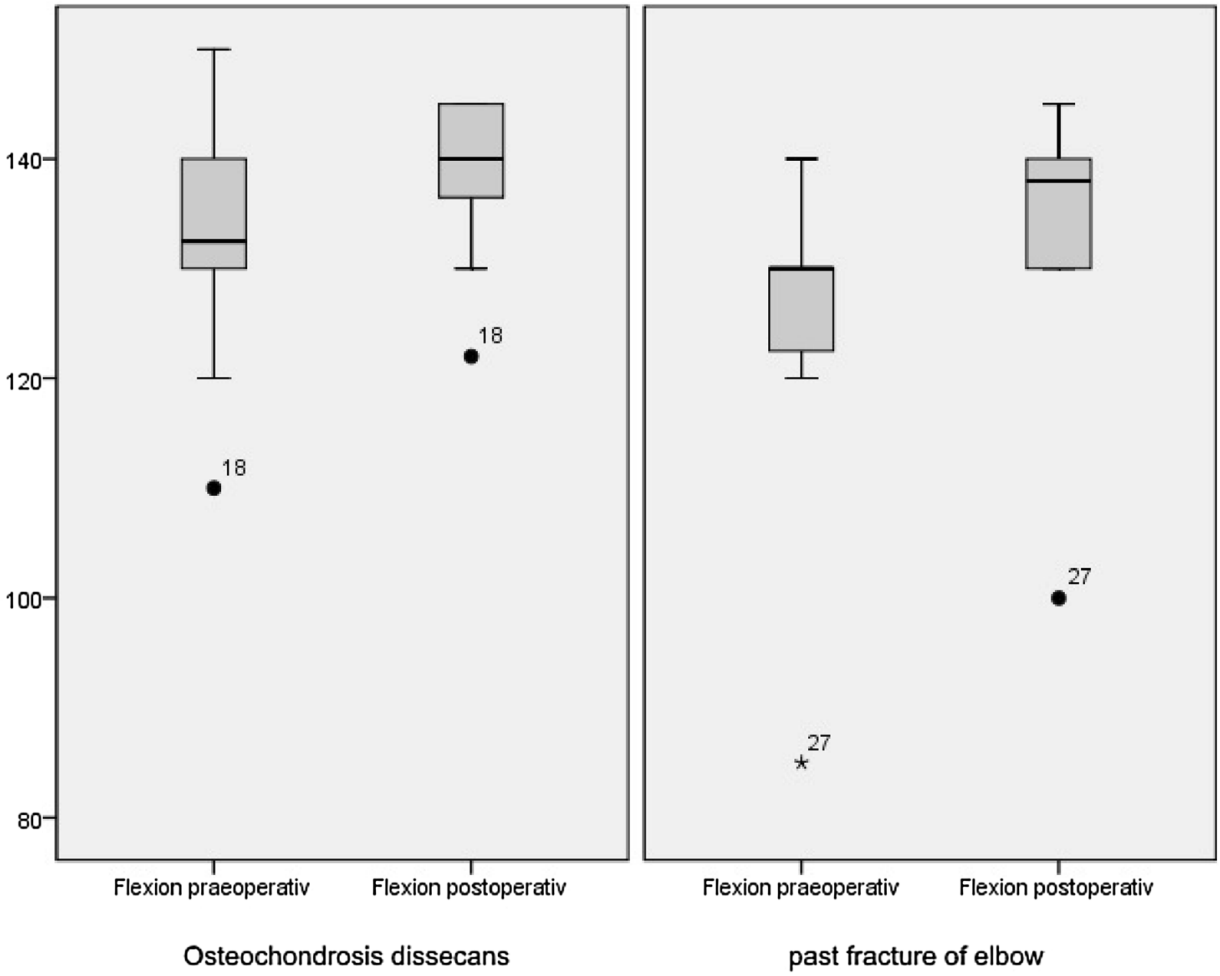
Boxplot of flexion before and after intervention left: OCD group 134° (SD: 11) to 139° (SD: 6), [*p* = .025]; right: arthrolysis group: 123° (SD: 18) to 132° (SD: 15), [*p* = .066]

The mean (SD) overall flexion improved from 131° (± 13.4) to 137° (± 9.5; *p* = .003) significant. Also in the subgroups the postoperative flexion was in the group PFE less (132° ± 15) than in the OCD group (139° ± 6). The increase of flexion improved in the OCD group significantly (from 134° [SD: 11] to 139° [SD: 6], *p* = .025), while the group PFE the improvement was not significant (from 123° [SD: 18] to 132° [SD: 15], *p* = .066) (Fig. 5). The mean preoperative and postoperative pronation was 84° ± 4.9 and 79.6° ± 5, respectively (*p* = .088) and the supination was 85° ± 9.8 and 83° ± 5 (*p* = .279).

**Fig. 6:**
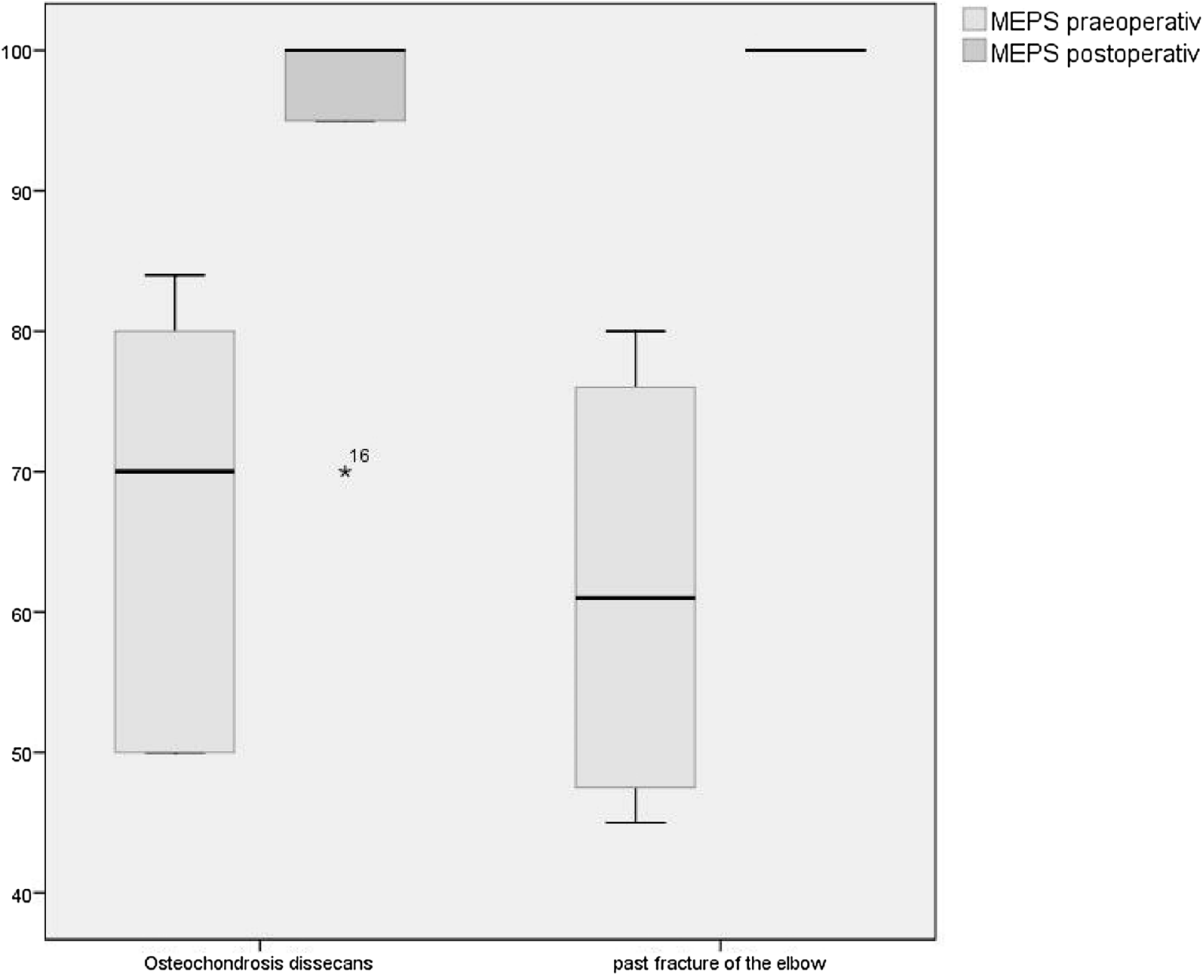
Boxplot of MEPS before and after intervention left: OCD group 67 (± 15) to 96 (± 8), [*p* = .027], right: arthrolysis group 61 (± 17) to 96 (± 9), [*p* = .068]

The mean (standard deviation) overall MEPS improved significant by elbow arthroscopy from 65 (± 15) to 96 (± 8; *p* = .005). In the OCD group, the MEPS was before the operation slightly higher (67 [± 15]) than in the PFE group (61 [± 17]), in which an increase of the MEPS was evaluated in both groups. The MEPS after the operation was in both subgroups with 96 (± 8 [OCD] and ± 9 [PFE]) equal, although the increase was just in the OCD group statistically significant (*p* = .027) (Fig. 6). 81.5% of all children and adolescents had excellent or good results according to the definition of the MEPS.

The OES and quick dash after the operations were with 5.5 and 96, respectively. The mean (SD) pain on visual analog scale was postoperative .2 (± .6), in which twenty-four (89%) children graduated with 0, one (4%) with 1 and two (7%) with 2. Complications in regard of damage of nerves or blood vessels were not observed (*n* = 0).

## Discussion

Although osteochondritis dissecans is the most common indication for elbow arthroscopy, arthroscopic arthrolysis is becoming more important. Nevertheless, elbow arthroscopy is still a less evaluated therapy option in the treatment of elbow diseases and is still subject of controversial debates. Especially with respect to children and adolescents, there are only a few studies, so the present study will give an overview of the treatment options and their results in this age group.

Osteochondral lesions which show no tendency of improvement under conservative therapy, a presence of free joint bodies or relevant cartilage damage (III and IV according to DiPaola) should be operated. Contrary to the current data from the knee joint, it is still controversially discussed whether conservative treatment or surgery should be preferred in case of open growth plates and higher OCD stage [14]. However, the status of the capitellar physis is an important prognosis parameter for the healing potential of an osteochondral lesion and is an essential component for the therapy concept.

The options of operative therapy are manifold and vary from open surgery with fixation of the loose fragment or osteochondral transplantation, to arthroscopic surgery with debridement of unstable lesions with or without bone marrow stimulation (e.g., microfracturing). Nevertheless, studies that evaluated surgical treatment in children and adolescents are rare. In the present study, an arthrolysis (20%), synovectomy (60%) and a removal of loose bodies (75%) combined with a removal of instable osteochondritis dissecans (25%) and microfracture (40%) were performed in case of a high grade and unstable OCD lesion. Both the ROM and the MEPS could be significantly improved in the group of OCD (Table 2). There are only a few studies showing good results after arthroscopic therapy of elbow diseases [2, 16]. Bojanic et al. evaluated 9 young teenage athletes (median age 15 years) and found in 8 excellent and in 1 case a good result after surgery [1]. No patient stopped participating in sports. Byrd et al. reported about 10 adolescent baseball players (average age 13.8 years) with arthroscopic surgery [3]. He could provide excellent rating scores with an intermediate follow-up; nevertheless, only 4 athletes returned to professional baseball. Micheli et al. reported in 2001 of 49 elbow arthroscopy in patients younger than 17 years, in which 55% (*n* = 27) were treated with an osteochondritis dissecans and 18% (*n* = 9) with an arthrofibrosis [13]. They could also show a good postoperative outcome measured with a modified Andrews Elbow Scoring System at 2-year follow-up. No complications were evaluated in this study. Ruth et al. reported about 12 patients aged under 17 years with osteochondral lesions of the capitellum [17]. In the study also a preoperative to postoperative increase in flexion–extension curve from 110 to 127 was seen and 11 of 12 patients had a pain relief.

Studies that evaluate arthroscopic arthrolysis in children and adolescents are significantly less and are mostly discussed in case studies, as in the present study. Micheli et al. performed 9 arthrolysis with arthroscopic joint debridement and release of flexion contractures, whereby 6 patients could be examined after 4.3 years postoperatively [13]. An improvement of the average loss of extension of 42° preoperative to 10° postoperative could be evaluated. The average maximum flexion was 109° preoperative, which improved to 140° postoperative. This corresponds to a slightly better improvement in range of motion compared to our study. One reason could be the reduced preoperative ROM compared to our collective.

It is known that using standardized portals and technique, the complication rate of elbow arthroscopy has a low complication rate. Also the experience of the surgeon has a huge impact on potential injuries of neurovascular structures [20]. The complication rate varies depending on the study between 0 and 14% [9, 13, 15]. It is known from the literature that palsy of the radial nerve is one of the more common complications and depends on the used approach, in which the middle or the high anterolateral approach is being considered the safest [7]. In the present study, also the middle anterolateral approach was used, which could explain the lack of complications with respect to vascular and nerve injuries.

Our study has some limitations, first the small number of involved children and adolescents and thus the low inferential statistical power. Furthermore, the retrospective design without a control group and the heterogeneity of indication for arthroscopic therapy are limitations.

## Conclusion

Elbow arthroscopy can be used in children and adolescents suffering from fracture sequelae such as contracture and pain and/or osteochondral lesions with good and very good postoperative results. Therewith it is possible to improve the postoperative range of motion and reduce pain, and can be regarded as a safe procedure in the hands of experienced surgeons.
